# Biogenic Synthesis of Zinc Oxide Nanoparticles Using *Citrullus colocynthis* for Potential Biomedical Applications

**DOI:** 10.3390/plants12020362

**Published:** 2023-01-12

**Authors:** Bushra Hafeez Kiani, Qudsia Ajmal, Nosheen Akhtar, Ihsan-ul Haq, Mostafa A. Abdel-Maksoud, Abdul Malik, Mohammed Aufy, Nazif Ullah

**Affiliations:** 1 Department of Biological Sciences, International Islamic University, Islamabad 44000, Pakistan; 2 Department of Biological Sciences, National University of Medical Sciences, Rawalpindi 46000, Pakistan; 3 Department of Pharmacy, Quaid-i-Azam University, Islamabad 45320, Pakistan; 4 Department of Botany and Microbiology, College of Science, King Saud University, Riyadh 11451, Saudi Arabia; 5 Department of Pharmaceutics, College of Pharmacy, King Saud University, Riyadh 11149, Saudi Arabia; 6 Department of Pharmaceutical Sciences, Division of Pharmacology and Toxicology, University of Vienna, 1010 Vienna, Austria; 7 Department of Biotechnology, Faculty of Chemical and Life Sciences, Abdul Wali Khan University, Mardan 23200, Pakistan

**Keywords:** biological activities, *Citrullus colocynthis*, enzyme inhibition assays, zinc oxide nanoparticles

## Abstract

Green nanoparticle synthesis is considered the most efficient and safe nanoparticle synthesis method, both economically and environmentally. The current research was focused on synthesizing zinc oxide nanoparticles (ZnONPs) from fruit and leaf extracts of *Citrullus colocynthis*. Four solvents (n-hexane, methanol, ethyl acetate, and aqueous) were used to prepare the extracts from both plant parts by maceration and extraction. Zinc acetate was used to synthesize the nanoparticles (NPs), and color change indicated the synthesis of ZnONPs. X-ray diffraction, UV spectroscopy, and scanning electron microscopy were used to study the ZnONPs. UV–visible spectroscopy revealed an absorbance peak in the 350–400 nm range. XRD patterns revealed the face-centered cubic structure of the ZnONPs. SEM confirmed a spherical morphology and a size range between 64 and 82 nm. Phytochemical assays confirmed that the complete flavonoid, phenolic, and alkaloid concentrations were higher in unrefined solvent extracts than in nanoparticles. Nanoparticles of *C. colocynthis* fruit aqueous extracts showed stronger antioxidant activity compared with the crude extracts. Strong antifungal activity was exhibited by the leaves, crude extracts, and nanoparticles of the n-hexane solvent. In a protein kinase inhibition assay, the maximum bald zone was revealed by nanoparticles of ethyl acetate extracts from leaves. The crude extracts and nanoparticles of leaves showed high cytotoxic activities of the n-hexane solvent, with LC_50_ values of 42.08 and 46.35, respectively. Potential antidiabetic activity was shown by the n-hexane (93.42%) and aqueous (82.54%) nanoparticles of the fruit. The bioactivity of the plant showed that it is a good candidate for therapeutic use. The biosynthesized ZnONPs showed promising antimicrobial, cytotoxic, antidiabetic, and antioxidant properties. Additionally, the in vivo assessment of a nano-directed drug delivery system for future therapeutic use can be conducted based on this study.

## 1. Introduction

The science of nanotechnology is quickly gaining popularity across the globe due to the physical and biochemical characteristics of nanomaterials [1]. Nanotechnology is anticipated to form the foundation for many of the twenty-first century’s most significant technical breakthroughs [2,3]. The formation of nanoparticles on small and large levels has been a significant contribution to nanoscience and is regarded as part of the upcoming industrial revolution [1]. Nanoparticles (NPs) with definitive forms, sizes, and shapes have been produced using a variety of traditional (chemical and physical) processes that are energy-intensive and hazardous to the environment due to the harmful compounds and chemicals used for their production. Compared with traditional techniques, the biological method (also known as green synthesis and the biogenic method) has been demonstrated to be simple, cost-effective, and eco-friendly with high stability [4,5]. The green synthesis of nanoparticles is the most preferred method because it provides high stability along with desired sizes and shapes. Moreover, nanoparticles produced by green synthesis are biocompatible because of the involvement of biomolecules as reducing agents. Furthermore, green synthesis methods are simple, easy, cost-effective, and environmentally friendly [6,7,8].

Among biogenic methods, plants are considered the best candidates. They appear to be appropriate for industrial nanoparticle production, as plants have a faster reproduction rate than other microorganisms such as bacteria, fungi, and algae [9,10]. Additionally, plant extracts contain different combinations of phytoconstituents (phytochemicals/active metabolites), such as enzymatic proteins, bio-alkaloids, isoprenoids, protein monomers, and bioflavonoids, which could contribute to bio-reduction as well as stabilize metallic NPs [11,12].

There are several uses for metallic and metal oxide nanoparticles in various fields, such as agriculture, catalysts, bio-labeling, and biomedical sciences [13,14,15]. Among the many noble metal nanomaterials, zinc oxide nanoparticles (ZnONPs) have been widely reported and used because of their size, various shape-dependent physicochemical properties, and biocompatible nature [16,17]. Sunblock is perhaps the most popular application of ZnONPs. These nanoparticles are utilized because they reflect UV light while remaining optically invisible in regular lighting. ZnONPs are under investigation for their potential to eliminate pathogenic microbes in ultraviolet-protecting products and packing-like textiles. ZnONPs are widely used for antimicrobial activities [18] and are applied in catalysis, labeling tags, sensors, medicine, and cosmetics [19,20]. Various studies have reported on the green synthesis of ZnONPs using different plant extracts [1,5,12]. Moreover, ZnONP is widely known as an ideal biomedical candidate, having biodegradable properties under both acidic and basic conditions in biological systems. Moreover, due to these inherent properties, including bactericidal and anticancer activities, ZnONP is a superior nanomaterial for nanodrug delivery [21].

The *Cucurbitaceae* family includes the desert plant *Citrullus colocynthis*, which has a broad genetic range. *C. colocynthis* has many medicinal properties, and it has a long history of being utilized as an herbal treatment in various regions [22,23]. According to phytochemical investigations, the biochemicals present in *C. colocynthis* include polyphenolic compounds, bio-alkaloids, bioflavonoids, and polysaccharides [23]. It possesses antimicrobial, antiulcer, anticancer, anti-inflammatory, anti-obesity, immune-stimulating, antioxidant, sedative, and wound-healing properties, and it can be used for the treatment of rheumatism, hemorrhoids, high blood sugar levels, and constipation [24]. Its roots are crushed and used as an ointment over swellings, and the paste is used for spleen and liver disease treatment [25].

Due to its vast biological potentialities, in the current research, leaf and fruit extracts of *C. colocynthis* were used to synthesize ZnONPs. The present study was aimed to examine and assess the medicinal properties (such as cytotoxic, anti-bacterial, and anti-cancer properties) and antioxidative characteristics of zinc oxide nanoparticles synthesized using methanol, n-hexane, ethyl acetate, and aqueous extracts from leaf and fruit extracts of *Citrullus colocynthis*. Additionally, the levels of all bio-alkaloids, bioflavonoids, and phenols were evaluated by comparing the crude extracts and ZnO nanoparticles of various solvents. To compare the effectiveness of ZnO nanoparticles against these enzymatic proteins, enzyme inhibition experiments, such as the protein kinase inhibition and alpha-amylase inhibition tests, were also conducted.

## 2. Results

### 2.1. Extract Recovery

The extract recovery was calculated for all four solvents of both parts. In total, eight samples were used (Table 1).

The liquid extract was divided into two parts. One half was kept to produce ZnONPs, and the other was stored to perform comparative assay analysis.

### 2.2. Synthesis of Zinc Oxide Nanoparticles

By continuously stirring a 10 mM ZnONP solution into the ethyl acetate, methanol, n-hexane, and aqueous extracts of fruits and leaves, ZnONPs were successfully synthesized. After 2 h of incubation, the color of the solution was changed to a light yellow hue, indicating that biogenic ZnONPs had been synthesized. The samples were placed on Petri dishes in very thin layers. In a drying oven, the plates were dried overnight at 80 °C. The nanoparticles were characterized using fine and dried powder.

### 2.3. Characterization of Zinc Nanoparticles

**Figure 1:**
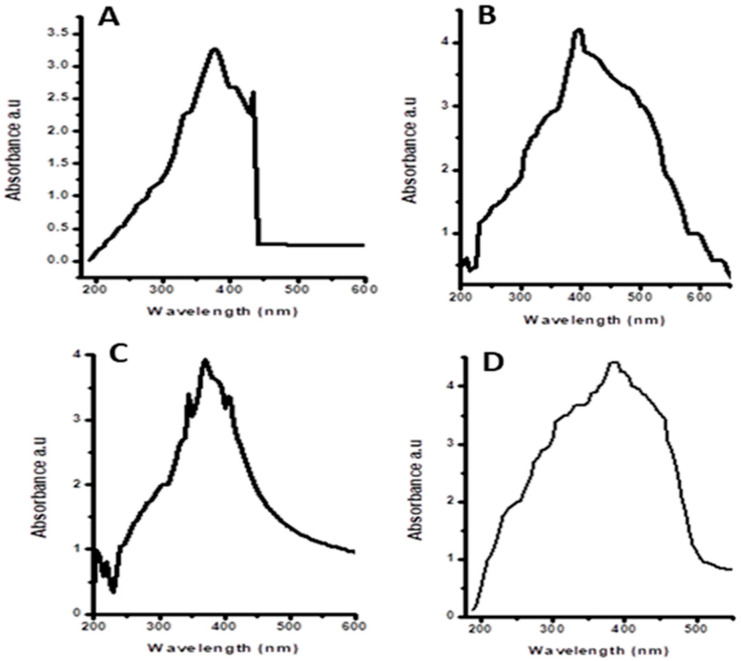
UV–visible absorption spectra of zinc oxide nanoparticles of leaves: (**A**) n-hexane, (**B**) ethyl acetate, (**C**) methanol, and (**D**) aqueous extracts.

**Figure 2:**
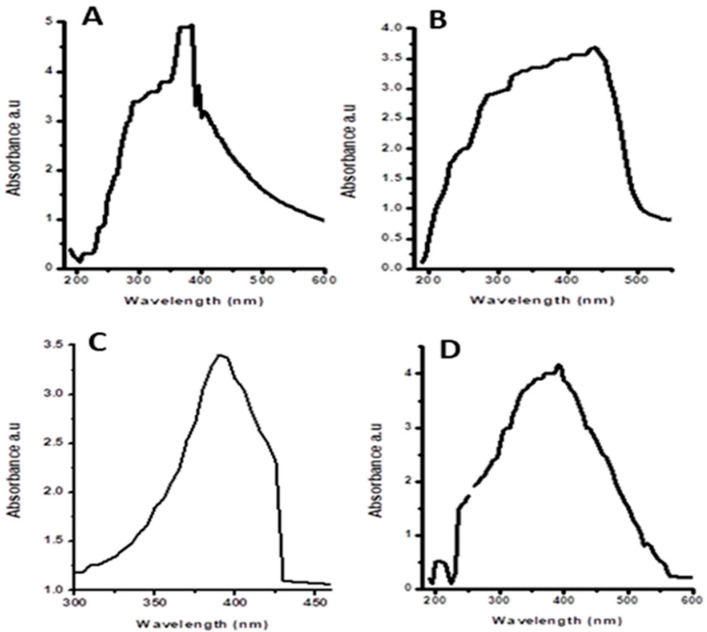
UV–visible absorption spectra of zinc oxide nanoparticles of fruits: (**A**) n-hexane, (**B**) ethyl acetate, (**C**) methanol, and (**D**) aqueous extracts.

UV–Vis spectroscopy was performed for the confirmation of ZNoNP production. UV absorbance peaks were observed within the range of 350–400 nm for synthesized ZnONPs from leaves (Figure 1) and fruits (Figure 2). The green synthesized ZnONPs showed characteristic absorption peaks of leaves and fruits at 357 nm, 367 nm, 356 nm, 360 nm, 370 nm, 373 nm, 365 nm, and 363 nm. Absorptions in the 356–370 nm wavelength range also confirmed that the spectrum of absorption was slightly blue-shifted in comparison with the ZnONPs’ bulk value (377 nm).

**Figure 3:**
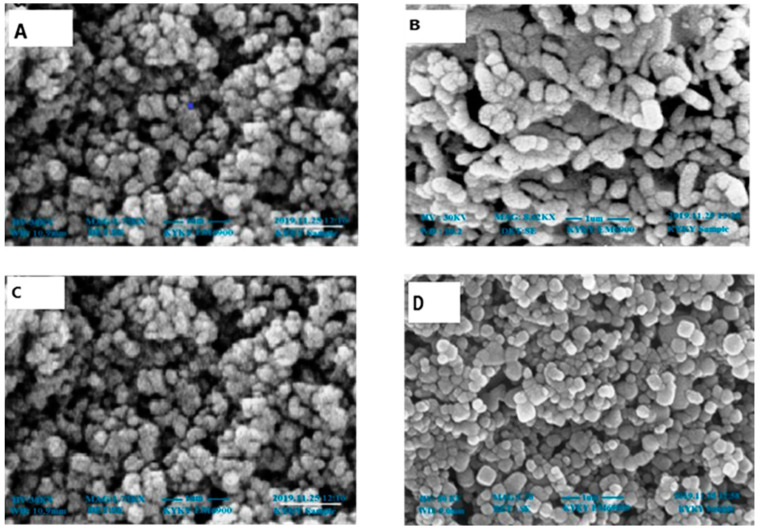
SEM images of zinc oxide nanoparticles of leaves: (**A**) n-hexane, (**B**) ethyl acetate, (**C**) methanol, and (**D**) aqueous extracts.

**Figure 4:**
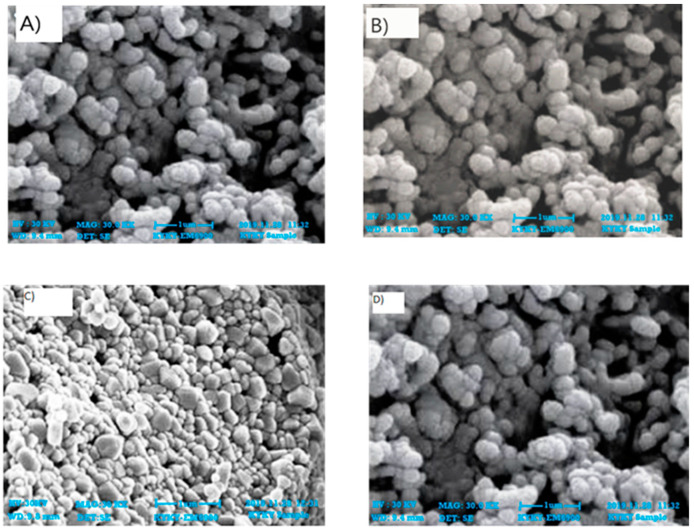
SEM images of zinc oxide nanoparticles of fruits: (**A**) n-hexane, (**B**) ethyl acetate, (**C**) methanol, and (**D**) aqueous extracts.

The scanning electron microscope findings are thought to be of great use in figuring out the shape, size, and particle-separating images of ZnONPs. The size of the ZnONPs prepared from leaves (Figure 3) and fruits (Figure 4) was found to be between 64 and 82 nm at 30 KV. Though the morphologies changed in some locations, spherical shapes predominated, and scanning electron microscope analysis showed a homogeneous population that was widely dispersed.

**Figure 5:**
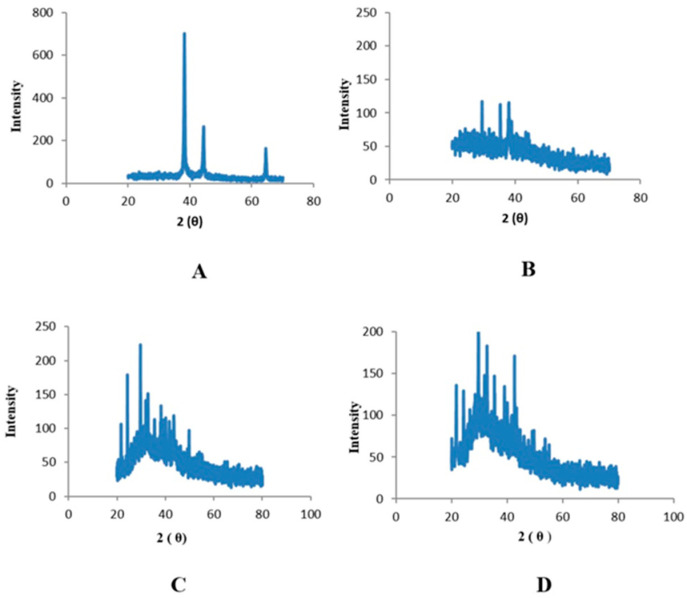
XRD peaks of zinc oxide nanoparticles of leaves: (**A**) n-hexane, (**B**) ethyl acetate, (**C**) methanol, and (**D**) aqueous extracts.

**Figure 6:**
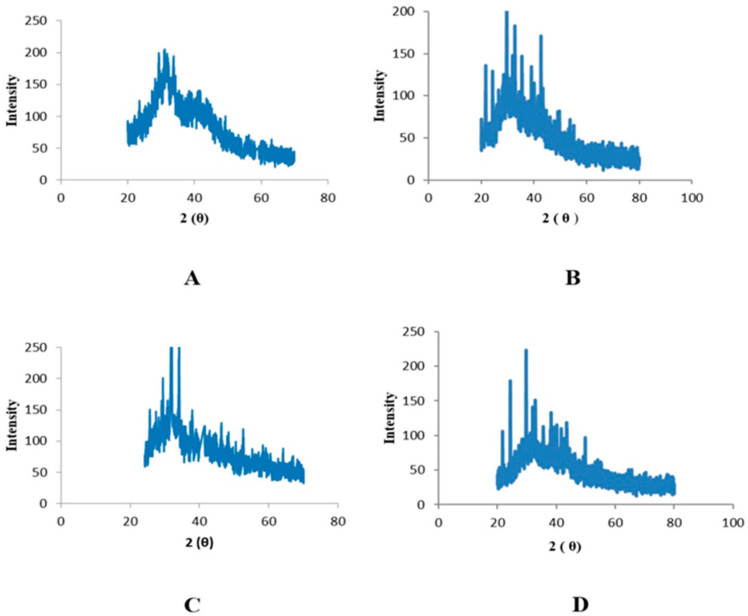
XRD peaks of zinc oxide nanoparticles of fruits: (**A**) n-hexane, (**B**) ethyl acetate, (**C**) methanol, and (**D**) aqueous extracts.

An X-ray diffractometer was used to prove the existence of nanoparticles and allowed for the analysis of their structural characteristics. The specifics of the crystal planes can be seen in the spectra. Zinc oxide nanoparticles associated with leaves displayed peaks at 25°, 30°, and 35° that were indexed as (100), (120), and (220) planes, respectively, and had 2θ values (Figure 5). Peaks with 2θ values detected in the zinc oxide nanoparticles derived from fruits were found at 25°, 30°, and 35°; these values were indexed as planes (121), (200), and (220), respectively (Figure 6).

### 2.4. Phytochemical Analysis

#### 2.4.1. Total Phenolic Contents

**Figure 7:**
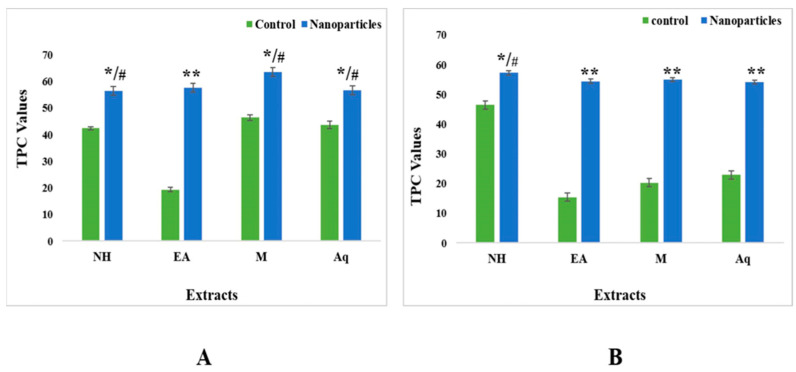
Total phenolic contents of control and nanoparticles from (**A**) leaf and (**B**) fruit extracts of *C. colocynthis*. Mean ± SD is expressed as */# *p* < 0.05, ** *p* < 0.01.

The results revealed the total phenol content in extracts, as measured in gallic acid equivalents per gram of dry weight of the plant. The measured results of the total phenol content (TPC) of the leaves in terms of both the control and nanoparticle fractions of the methanolic extracts showed higher values (46.38 GAE/g DW and 63.45 GAE/g DW, respectively) compared with the other three extracts, i.e., n-hexane (42.36 GAE/g DW and 56.46 GAE/g DW, respectively), ethyl acetate (19.35 GAE/g DW and 57.67 GAE/g DW, respectively), and water (46.38 GAE/g DW and 56.774 GAE/g DW, respectively) (Figure 7).

Control and nanoparticle fractions of the n-hexane extracts of fruits showed higher values (46.49 GAE/g DW and 57.29 GAE/g DW, respectively). The lowest value for aqueous extract nanoparticles was 54.15 GAE/g DW (Figure 7).

#### 2.4.2. Total Flavonoid Contents

**Figure 8:**
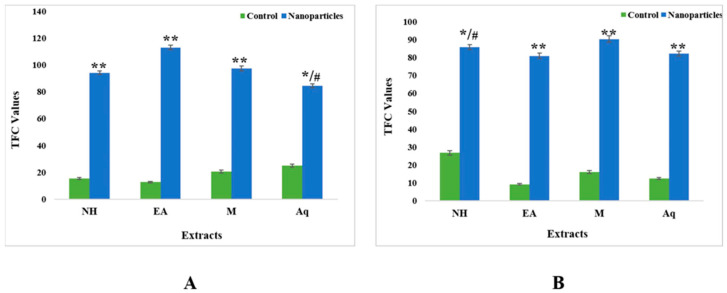
Total flavonoid contents of control and nanoparticles from (**A**) leaf and (**B**) fruit extracts of *C. colocynthis*. Mean ± SD is expressed as */# *p* < 0.05, ** *p* < 0.01.

The total flavonoid content was calculated in terms of quercetin equivalents. The results for the control extracts’ leaf fractions revealed the total flavonoid contents (TFC) to be in the range from 12.74 QE/g DW to 25.02 QE/g DW. The aqueous extract showed the highest TFC value (25.02 QE/g DW), and the ethyl acetate extract showed the lowest value (12.75 QE/g DW). For nanoparticles, the results were the complete opposite, as the ethyl acetate extract showed the highest value (113.02 QE/g DW) compared with the n-hexane (94.18 QE/g DW), methanol (97.48 QE/g DW), and aqueous (84.38 QE/g DW) extracts (Figure 8).

For fruits, the control fractions of ethyl acetate had the lowest TFC value of 9.32 QE/g DW, whereas for nanoparticles, the methanolic extracts had the maximum TFC value of 90.27 (Figure 8).

#### 2.4.3. Total Alkaloid Contents

**Figure 9:**
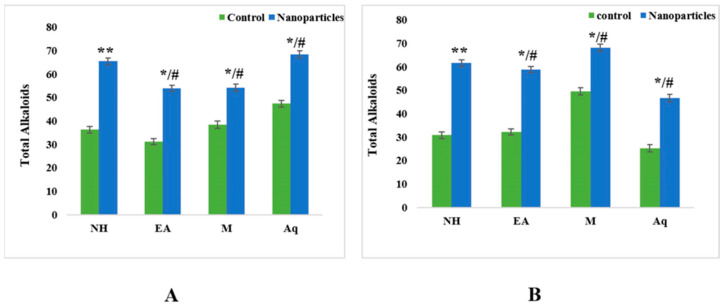
Total alkaloid contents of control and nanoparticles from (**A**) leaf and (**B**) fruit extracts of *C. colocynthis*. Mean ± SD is expressed as */# *p* < 0.05, ** *p* < 0.01.

The total alkaloid levels in control extract leaf fractions ranged from 31.23 mg to 47.34 mg. The greatest alkaloid content was found in the aqueous extract (47.34 mg), and the lowest alkaloid content was found in the ethyl acetate extract (31.23 mg). In nanoparticles, the same trend was observed, with the aqueous extract having the maximum alkaloid content (68.64 mg) and the ethyl acetate extract having the smallest value (53.65 mg) compared with the n-hexane (65.45 mg) and methanol (54.19 mg) extracts (Figure 9).

For fruits, the control fractions of ethyl acetate had the lowest alkaloid content of 25.31 mg, whereas for nanoparticles, the methanolic extracts had the maximum alkaloid content of 69.22 mg (Figure 9).

### 2.5. Biological Activities

#### 2.5.1. Antioxidant Assay

**Figure 10:**
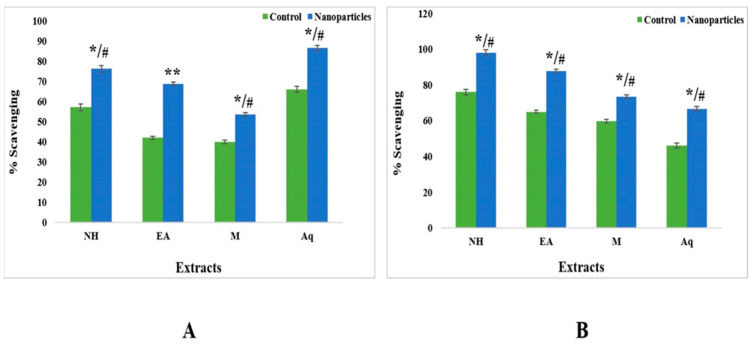
Comparative analysis of control and zinc oxide nanoparticles from (**A**) leaf and (**B**) fruit extracts of *C. colocynthis*. Mean ± SD is expressed as */# *p* < 0.05, ** *p* < 0.01.

Regarding the proportion of DPPH scavenging for control fractions in leaves, all four extracts showed a minimum antioxidant activity with IC_50_ > 200 µg/mL. In comparison, nanoparticles showed the highest activity in the aqueous extract (86% with IC_50_ 13.35), followed by the n-hexane (IC_50_ 25.93) and ethyl acetate (IC_50_ 28.42) extracts (Figure 10).

The crude n-hexane, ethyl acetate, methanol, and aqueous extracts of fruits showed a minimum percentage inhibition with IC_50_ > 200. However, nanoparticles of the n-hexane extract showed the highest IC_50_ value of 24.05, followed by ethyl acetate (IC_50_ 26.47) and methanol (IC_50_ 32.68) extracts (Figure 10).

#### 2.5.2. Antibacterial Assay

**Figure 11:**
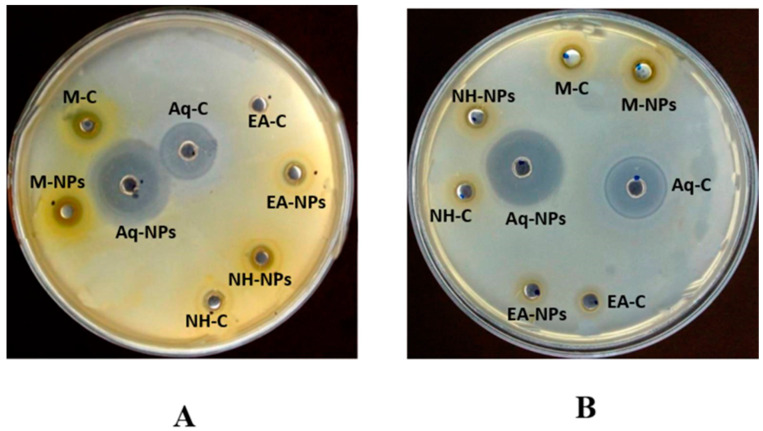
Anti-bacterial activity of ZnONPs and crude extracts of different solvents of C. *colocynthis.* (**A**) From leaves; (**B**) from fruits. AqNPs = aqueous nanoparticles; AqC = aqueous control; MNPs = methanol nanoparticles; MC = methanol control; NHNPs = n-hexane nanoparticles; NHC = n-hexane control; EANPs = ethyl acetate nanoparticles; EAC = ethyl acetate control.

Both crude extracts and nanoparticles of *C. colocynthis* (leaf and fruit parts) were found to have anti-bacterial activity. The crude aqueous extract of leaves showed the highest level of activity, i.e., 28 ± 0.27 (MIC = 11.1) for *E.coli*, followed by 21 ± 0.45 (MIC = 33.3) for *B. subtilis* and 13 ± 0.45 (MIC = 100) for *K. pneumoniae*. In the case of nanoparticles, significant activity was shown by the aqueous extract, with 39 ± 0.43 (MIC = 3.7) against *E.coli*, 3.4 ± 0.19 (MIC = 3.7) against *Bacillus subtilis* and 24 ± 0.32 (MIC = 33.3) against *Klebsiella pneumoniae.* This was followed by n-hexane, with 39 ± 0.43 (MIC = 3.7) against *K. pneumoniae* and 28 ± 0.41 (MIC = 3.7) for *B. subtilis.* The minimum activity was observed against *P. aeruginosa* and *S. aureus* (Figure 11 and Table 2).

For fruits, methanol demonstrated the strongest antimicrobial effects among the crude extracts, i.e., 25 ± 0.19 (MIC = 33.3) against *E. coli*. In nanoparticles, significant activity was seen in the aqueous extract, i.e., 29 ± 0.35 (MIC = 33.3) against *B. subtilis*, followed by methanol 18 ± 0.15 (MIC = 100) against *E. coli*. The minimum activity was recorded against *P. aeruginosa* and *S. aureus* (Figure 11 and Table 2).

#### 2.5.3. Antifungal Assay

The crude extracts and nanoparticles of the leaf extracts of the polar solvents showed good activity against *F. solani,* i.e., methanol (9 mm and 12 mm, respectively) and aqueous (10 mm and 11 mm, respectively) extracts. Nonpolar solvents showed less activity against all tested strains (Table 3).

In the case of fruits, good activity was observed by crude extracts against *A. fumigatus* in the order of methanol (12 mm), DW (11 mm), n-hexane (10 mm), and ethyl acetate (10 mm). The extracts also showed maximum activity against *F. solani,* i.e., methanol (13 mm), aqueous (11 mm), ethyl acetate (12 mm), and n-hexane (13 mm) extracts. For nanoparticles, polar extracts showed maximum zones of inhibition against *A. fumigatus* and *F. solani,* i.e., methanol (13 mm and 11 mm, respectively), aqueous (12 mm and 13 mm, respectively), ethyl acetate (11 mm and 12 mm, respectively), and n-hexane (11 mm and 11 mm, respectively) extracts. The *Aspergillus flavus* and *Mucor* species showed no or very little action. (Table 3). The results showed that the NPs synthesized from the aqueous extract of leaves and the methanolic extract of fruits demonstrated significant efficacy against *Aspergillus* flavus compared with the other extracts. Similarly, the NPs of methanol and ethyl acetate extracts demonstrated significant antifungal activity against *Aspergillus fumigatus and Fusarium solani* compared with the extracts alone.

#### 2.5.4. Cytotoxicity Assay

The cytotoxic assay results showed that at a concentration of 200 µg/mL, the NPs of leaves from the n-hexane and ethyl acetate extracts and NPs of fruits from the n-hexane extracts showed significantly less toxicity than the crude extracts. At 100 µg/mL, the NPs of leaves from the n-hexane and ethyl acetate extracts and the NPs of fruits from the methanol extract exhibited less toxicity than the crude extracts. Significantly less toxicity was observed in the NPs of the ethyl acetate and methanol extracts at a 50 µg/mL concentration from fruit extracts.

The crude extracts of leaves showed a significant cytotoxic impact, but the n-hexane extract was considered to be more effective, with an LC50 value of 42.41 µg/mL. This was followed by the ethyl acetate extract, which had an LC50 value of 72.92 µg/mL. The methanol and aqueous extracts showed the least activity, with LC50 values > 200 µg/mL. The LC50 values were higher for nanoparticles, i.e., the n-hexane extract was most active, with an LC50 value of 46.84 µg/mL; followed by the ethyl acetate extract, with an LC50 value of 110.45 micrograms per milliliter; the methanol extract, with an LC50 value of 150.8 µg/mL, and the aqueous extract, with an LC50 value of less than 200 µg/mL (Table 4).

For the crude extracts of fruits, the n-hexane extract showed a significant LC50 value of 140.6 µg/mL, succeeded by the ethyl acetate extract, with an LC50 value of 170.6 µg/mL. The other extracts showed minimum activity, with LC50 values > 200 µg/mL. The maximum value for nanoparticles was found for the n-hexane extract (LC50 value 130.5 µg/mL), followed by the ethyl acetate extract (LC50 value 146 µg/mL) and the methanol extract (LC50 value 185.8 µg/mL). The aqueous extract showed no activity (Table 4).

### 2.6. Enzyme Inhibition Assays

#### 2.6.1. Protein Kinase

The nanoparticles showed strong bactericidal effects against different bacterial strains. For leaves, the control fraction of the n-hexane extract showed no activity compared with the nanoparticles’ bactericidal effect. For the methanolic extracts, the control showed anti-tumor activity and the nanoparticles showed both bactericidal and anti-tumor activities. For the ethyl acetate and aqueous extracts, both control and nanoparticles presented bactericidal effects (Table 5).

For fruits, the control extracts of n-hexane and ethyl acetate showed no activity, but the other solvents showed bactericidal and anti-tumor activities along with their nanoparticles. The n-hexane and ethyl acetate nanoparticles also showed anti-tumor and bactericidal activities (Table 5).

#### 2.6.2. Alpha-Amylase Assay

**Figure 12:**
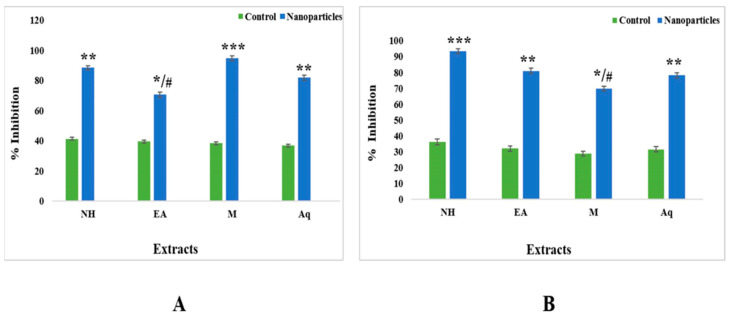
Alpha-amylase inhibition of nanoparticles and control from leaves of C. colocynthis: (**A**) fruits; (**B**) leaves. Data are expressed as the mean ± SD. */# *p* < 0.05, ** *p* < 0.01 *** *p* < 0.001.

To analyze the antidiabetic properties of plants, an alpha-amylase assay was carried out. The findings are expressed as percent alpha-amylase, and *C. colocynthis* showed significant alpha-amylase inhibition capability. The total antidiabetic activity of the control fractions of fruits ranged from 36.19% in the n-hexane extract to 28.86% in the aqueous extract. For nanoparticles, the methanol extract showed 93.42% activity, followed by the n-hexane (83.05%), ethyl acetate (69.86%), and aqueous (82.54%) extracts (Figure 12).

Leaf control fractions range from 41.41% for the n-hexane extract, 39.5% for the ethyl acetate extract, 38.41% for the methanol extract, and 36.79% for the aqueous extract. For nanoparticles, the n-hexane extract showed 91.3% inhibition, the ethyl acetate extract showed 79.41% inhibition, the aqueous extract showed 79.5% inhibition, and the methanol extract showed 69.7% inhibition (Figure 12).

## 3. Discussion

One of the most commonly used metal oxide nanoparticles is zinc oxide because of its biocompatibility, low toxicity, and low cost. It has recently become a reliable alternative for applications in optics, the electric packaging of food, and pharmaceuticals. ZnONPs trigger cell apoptosis via the high generation of reactive oxygen species and the release of zinc in ionic forms. The biogenic synthesis of ZnONPs seems to be easy, safe, more sustainable, and more ecologically friendly than physical and chemical procedures employing plant extracts. These biologically synthesized NPs are used in various biological applications because they have high biological activity [26].

In the current study, methanol, n-hexane, aqueous, and ethyl acetate extracts of *Citrullus colocynthis* leaves and fruits were used to synthesize ZnONPs, and a comparison of different pharmacological properties (antimicrobial, anti-tumor, antioxidant, and cytotoxic) was carried out. When the methanol, n-hexane, aqueous, and ethyl acetate extracts were treated with a zinc acetate solution, the color of the solution was changed to light yellow and confirmed the synthesis of ZnONPs [27]. Different parts of *Citrullus colocynthis* are used to treat different diseases [28]. The fruit’s pulp has caffeic acid supplements, gum, colocynthium, pectic acid, calcium, lignin, and magnesium. The water present in the pulp contains three essential acids: fatty acids, linoleic acids, and oleic acids. Studies conducted on albino rats have shown the anti-inflammatory activity of the ethanolic extracts of roots. It has been discovered that quercetin and kaempferol are present in its flowers and leaves. Due to this, the plant has significant antimicrobial properties. The powder is effective against Gram-negative and Gram-positive bacterial species, even at a minimum quantity of 0.6–1 g [29,30]. ZnONPs can be characterized using UV spectroscopy, XRD, and scanning electron microscope examination, as reported by [31]. In this study, the green synthesized ZnONPs showed characteristic absorption peaks of leaves and fruits at 357 nm, 367 nm, 356 nm, 360 nm, 370 nm, 373 nm, 365 nm, and 363 nm. According to past research, these absorption peaks could be attributed to ZnO’s inherent band-gap absorption because of the electron transitions between the valence band and the conduction band (O2 pZn3 d) [32]. The good absorption of ZnONPs in the ultraviolet region demonstrates their suitability for medical applications, such as sunscreen protectors or antiseptic ointments [33]. These spectral analysis outcomes are consistent with those of many other studies in which the absorption peak of phyto-fabricated ZnO nanoparticles was between 280 nm and 400 nm. This absorption range is crucial for nanoparticle applications in the biological and pharmaceutical fields [26,34] (Figure 1 and Figure 2).

The morphology of phyto-fabricated ZnONPs visualized under SEM showed the size and agglomeration of nanoparticles. The morphological study of ZnONPs showed that the size of ZnONPs ranged from 64 to 82 nm, with a spherical shape (Figure 3 and Figure 4), which was in accordance with earlier reports by [12,26,35,36,37]. The crystalline nature of zinc nanoparticles was confirmed by XRD, thus confirming that the zinc nanoparticles formed in the present study were nanocrystals, as evidenced by the peaks at 20°, 25°, 30°, and 40° corresponding to different planes (Figure 5 and Figure 6).

These findings showed that during the phyto-fabrication of ZnONPs, the metabolites in the plant extracts worked as capping/stabilizing agents to produce a metal oxide, which is in line with other researchers’ findings [9,36].

Dried plant extracts of *Citrullus colocynthis* were used for phytochemical analysis. Phenolic compounds are plant compounds with redox properties that can result in antioxidant activity [22]. Our phytochemical analysis revealed that the measured values of the total phenolic contents (TPC) of both the control and nanoparticle fractions of the methanolic extract of leaves showed higher values (46.38 and 63.45, respectively) compared with the other three extracts (Figure 7). In the case of fruits, the control and nanoparticle fractions of n-hexane extract showed the highest values (46.49 and 57.29, respectively) (Figure 7). The dissolution of endogenous compounds in plants results from extraction processes and solvents [37]. Additionally, plant compounds are both polar and nonpolar in nature. Phenolic compounds are much more soluble in polar and organic solvents because of their hydroxyl groups [38]. The authors of [39] also assessed a *S. nigrum* water extract’s total phenol content, which was 0.704 mg GAE/g fresh weight [39,40], and recorded total phenol content of 180.64 6.51 mg GAE/g using a methanol extract of *Cassia tora* [40]. A total phenol content of 3.6 ± 0.089 mg GAE/g dry weight was reported by [41] in an n-hexane extract of *P. oleracea* [41]. Slight differences have been observed in the phenol content reported in different studies. Different plant species, carotenoids, duration and quantity of sugars, geographic variance, ascorbic acid, and extraction techniques could all contribute to this alteration [42].

The total flavonoid contents (TFC) results for the leaf fractions of control extracts were between 12.74 QE/g dry weight and 25.02 QE/g dry weight. The aqueous extract showed the highest TFC value (25.02 QE/g dry weight), and the ethyl acetate extract showed the lowest value (12.75 QE/g dry weight). We observed unique results for nanoparticles, with opposite results: the ethyl acetate extract showed the highest value (113.02 QE/g dry weight) compared with the n-hexane (94.18 QE/g dry weight), methanol (97.48 QE/g dry weight) and aqueous (84.38 QE/g dry weight) extracts (Figure 8). For fruits, the control fractions of ethyl acetate expressed a minimum value of 9.32 QE/g dry weight, whereas for the nanoparticles, the methanolic extract displayed the greatest TFC value of 90.27 QE/g dry weight. (Figure 8). Flavonoids have antioxidant functionality and are secondary metabolites whose potency depends on the number and placement of hydroxyl groups [43]. The authors of [44] found that the methanol extract of *C. tora* contained a total flavonoid content of 21.53 mgQE/g dry weight [44]. The authors of [45] showed that a water extract of *S. nigrum* contained a TFC of 0.64 QE/g dry weight, and a methanol extract of *Portulaca oleracea* contained a TFC of 49.2 ± 3.4 mgQE/g dry weight. As mentioned in the literature, genetic diversity, seasonal or yearly variations, and environmental and biological factors affect the content of flavonoids in vegetables [20]. Here, the results for the leaf fraction of the control extracts’ total alkaloids ranged from 31.23 mg to 47.34 mg. The aqueous extract showed the highest alkaloid content in both the control (47.34 mg) and nanoparticles (68.64 mg). For fruits, the control fractions of ethyl acetate showed a minimum value of 25.31 mg, whereas the methanolic extract in the context of nanoparticles displayed the highest amount of alkaloid concentration, i.e., 69.22 (Figure 9).

Infections caused by bacteria pose serious threats to human health. Due to rising antibiotic resistance in bacteria and the advent of novel strains, researchers have concentrated on nanoparticles obtained from metals and metals oxides that have anti-bacterial properties [12]. Because of their unique physicochemical characteristics and large surface areas, ZnONPs have been widely researched as anti-bacterial agents. They are also non-toxic and compatible with the human body [26]. Several studies have reported that the possible anti-bacterial mechanisms of ZnONPs involve three different steps. Firstly, the production of ROS causes oxidative stress, the disruption of the cell membrane, and DNA damage, thus destroying bacterial cells. Secondly, when ZnONPs dissolve, Zn^2+^ ions are released. These ions lead to the death of bacteria by interacting and destroying the integrity of the cell membrane, cytoplasm, and nucleic acid. Lastly, the plasma membrane is harmed, and intracellular components leak due to direct electrostatic interactions among ZnONPs and the cell membranes of bacteria [46]. In our antimicrobial assay, we observed very interesting results. Crude extracts of *Citrullus colocynthis* showed increased functionality for all bacterial strains tested compared with ZnONPs, which showed the least anti-bacterial activity. In the case of leaves, the highest anti-bacterial activity of the different crude extracts was found in the aqueous extract, while in fruits, the highest activity was recorded in the methanolic crude extract (Table 2). All plant extracts containing ZnONPs showed significant antifungal activity at all tested concentrations. The crude extracts and nanoparticles of the leaf extracts of polar solvents showed significant activity against *F. solani,* i.e., methanol (9 mm and 12 mm, respectively) and aqueous (10 mm and 11 mm, respectively) extracts. Nonpolar solvents showed less activity against all tested strains. In the case of fruits, significant activity was observed against *A. fumigatus* in the crude methanol extract, followed by the DW, n-hexane, and ethyl acetate extracts. The methanol extract also showed maximum activity against *F. solani* by, followed by the n-hexane, aqueous, and ethyl acetate extracts. For nanoparticles, the polar extracts showed maximum zones of inhibition against *A. fumigatus* and *F. solani,* i.e., the methanol extract followed by the aqueous, ethyl acetate, and n-hexane extracts. The *A. flavus* and *Mucor* species showed no or very little action. (Table 3). According to these findings, crude extracts may be a reliable source of antimicrobial compounds that demand additional therapeutic studies.

In a previous study, the hydrothermal synthesis of ZnONPs from *Ceropegia candelabrum* leaf extracts produced nanoparticles with good purity, a hexagonal wurtzite form, and diameters of 12–35 nanometers [5]. The biosynthesized ZnONPs showed anti-bacterial potential against microbial strains such as *S. aureus*, *B. subtilis*, *E. coli*, and *S. typhi* at 100 µg/mL. Another group of researchers synthesized ZnONPs from *C. religiosum* leaf extracts to investigate their anti-bacterial activity [36]. With minimum inhibitory concentrations ranging from 4.8 to 312.5 µg/mL-1, the bio-fabricated ZnONPs from *C. colocynthis* (L.) Schrad (fruit, seed, and pulp) significantly inhibited Gram-positive strains such as *Bacillus subtilis* and *Staphylococcus aureus* and Gram-negative strains such as *P. aeruginosa* and *Escherichia coli* bacteria [47].

Due to their ability to scavenge 2,2,diphenyl-1-picrylhydrazyl free radicals, ZnONPs change an unstable, deep violet-colored methanolic solution of 2,2,diphenyl-1-picrylhydrazyl into a stable, pale-yellow solution, which is the basis for their antioxidative capability [48,49]. To evaluate antioxidative action in the studied samples, a 2,2,diphenyl-1-picrylhydrazyl free radical scavenging assay was used. This assay can be used to determine the antioxidant properties of vegetables, wheat, edible seed oils, flours, herbs, and conjugated linoleic acids [50,51]. This method does not involve enzymes and can provide essential data on compounds’ capability to scavenge free radicals [52]. Significant DPPH scavenging activity was observed in the ZnONPs of plant extracts compared with control extracts at all tested concentrations. Among all tested samples, the proportion of DPPH scavenging for control fractions in leaves in all four extracts showed minimum antioxidant activity, with IC_50_ > 200 µg/mL. In comparison, nanoparticles showed the highest activity with the aqueous extract, succeeded by the ethyl acetate and methanol extracts (Figure 10). Our findings are consistent with various reports in which an enhanced antioxidative action was noted when the concentration of zinc oxide nanoparticles was increased. It was reported that green synthesized ZnONPs exhibited significant antioxidative properties due to their charge density and surface capping materials [53]. Another study reported that ZnONPs biologically prepared from extracts of *Sambucus ebulus* leaves expressed free radical scavenging activity and revealed that the biosynthesized ZnONPs’ antioxidant properties could be enhanced by the existence of metal or Zn ions in the structure [49]. In another study, aqueous fruit extracts of *Myristica fragrans* were used to create zinc oxide nanoparticles, which were found to have great free radical scavenging activity [15].

The authors of [54] proposed that the *Brine shrimp* cytotoxicity assay is useful for analyzing pharmaceutical activities of plant extracts. The results of the cytotoxic assay in the present study revealed a significant effect in leaf crude extracts: the n-hexane extract was considered the most effective, followed by the ethyl acetate extract, with the methanol and aqueous extracts showing the least activity. In the case of NPs, the n-hexane extract expressed the most activity, followed by the methanol, aqueous, and ethyl acetate extracts. In the case of the crude extracts of fruits, the n-hexane extract was found to be highly active, followed by the ethyl acetate extract. The maximum value for nanoparticles was observed for the n-hexane extract, followed by the methanol, ethyl acetate, and aqueous extracts, which showed no activity (Table 4). The fundamental cytotoxic mechanism of ZnONPs is the release of zinc ions that are intracellularly dissolved along with the induction of reactive oxygen species. This activity is due to a binary response consisting of the cell’s pro-inflammatory reactions against ZnONPs and the basic surface property of the nanoparticle, which allows ZnONPs to act as an oxidation–reduction system [55].

Different enzyme inhibition assays were performed to further assess the properties of *Citrullus colocynthis.* During the protein kinase inhibition assay, nanoparticles showed strong bactericidal effects against bacterial strains. For leaves, the control fraction of the n-hexane extract showed no activity compared with the nanoparticles’ bactericidal effect. For the methanolic extracts, the control showed anti-tumor activity and nanoparticles showed both bactericidal and anti-tumor activities. Both the control and nanoparticles presented bactericidal effects for the ethyl acetate and aqueous extracts. For fruits, the control extracts of n-hexane and ethyl acetate showed no activity, but the other solvents showed bactericidal and anti-tumor activities along with their nanoparticles. The n-hexane and ethyl acetate nanoparticles also revealed anti-tumor and bactericidal activities (Table 5).

An alpha-amylase assay was conducted to test the plant’s antidiabetic potency, and the extracts of *C. colocynthis* showed significant alpha-amylase inhibition capability. The control fractions of leaves was 41.41% for the n-hexane extract, followed by the ethyl acetate, methanol, and aqueous extracts. The n-hexane extract showed 88.3% inhibition for nanoparticles, followed by the methanol, ethanoate, and aqueous extracts. The total antidiabetic activity of the control fractions of fruits ranged from 36.19% in the n-hexane extract to 28.86% in the aqueous extract. For nanoparticles, the n-hexane extract showed 93.42% activity, followed by the methanol, ethyl acetate, and aqueous extracts (Figure 12).

## 4. Material and Methods

### 4.1. Collection of Plant Samples

*Citrullus colocynthis* (L.) Schrad. parts (foliage and fruits) were collected from the Cholistan desert, situated in the district Bahawalpur, Pakistan, and experts recognized it at the Department of Plant Sciences, Quaid-i-Azam University, Islamabad, as *Citrullus colocynthis* (L.) Schrad. The fruit samples were collected when they completely ripened, the color turned yellow, and the leaves were fully green. Leaves were collected from the top end of the vein. The plant perennially grows from the root, and its aerial part dies in winter and grows again in spring. The fruits and leaves were collected when the age of the vein was approximately five months. The samples were stored in the herbarium of the department for future reference (ACC 543217). Leaves and fruits were separated, cleaned of dirt, and left to dry in the shade. A mortar and pestle were used to grind these dehydrated leaves and fruits. For future usage, the powdered sample was kept separate.

### 4.2. Plant Extract Formulation

Dried *C. colocynthis* samples were used to make extracts via a simple maceration procedure described by [53]. The used solvents were non-polar or polar, i.e., ethyl acetate, n-hexane, methanol, and aqueous (DW). First, 100 g of the plant powder was soaked in 600 mL of each solvent for three days at room temperature (23–27 °C). The extraction process was enabled by ultra-sonication in an ultrasonic bath for 30 min at 23–27 °C. The extracts were filtered and separated into 2 parts for every solvent using Whatman filter No. 1 paper. This technique was performed two times, and the resulting extracts were subsequently mixed and reduced via evaporation on a vacuum rotary evaporator followed by drying in a vacuum dryer at 45°C to produce the finished crude extracts. The percentage of extract recovery was used to indicate the yield of specific *C. colocynthis* extracts in various solvent systems. The formula to determine the crude extract’s percent recovery is as follows:

% Extract recovery (%*w*/*w*) = (A/B) × 100

A = dried extract weight.

B = plant material (powdered) weight.

Stock solutions at concentrations of 4 mg/mL and 20 mg/mL were synthesized in DMSO. Solvents were added to the appropriately labeled Eppendorf tubes after the carefully weighed quantities were poured. To create clear stock solutions, the samples were ultimately ultra-sonicated.

### 4.3. Synthesis of Zinc Oxide Nanoparticles (ZnONPs)

Zinc oxide nanoparticles were produced according to the method described by [56] with some modifications. The plant extracts (50 g) were heated in a breaker at 50–60 °C for 30 to 40 min using a hot plate. The heated extracts were continuously stirred for 2 h after adding zinc acetate (5 g) to synthesize ZnONPs. After 2 h, a color shift was seen, and the extract was cooled. A thin extract coating was applied to Petri dishes and left to dry overnight at 80 °C. This finely dried powdered sample was prepared for the characterization process. The same method was used to prepare extracts from all solvents.

### 4.4. Characterization of Zinc Oxide Nanoparticles

#### 4.4.1. UV–Visible Spectroscopy

UV–Vis spectroscopy is an extensively employed tool for the characterization of NPs [57] that follows the Lambert–Beer law [58]. Here, the synthesized ZnONPs were characterized by measuring their optical density at the wavelength range of 350–400 nm with a resolution of 1 nm.

#### 4.4.2. Scanning Electron Microscopy Analysis (SEM)

On a micrometer to nanoscale scale, the nanoparticles’ structure and diameter were examined using SEM (KYKY-EM6900) [59]. After applying a sample solution drop on a grid equally coated with carbon, the ZnONPs were assessed by dehydrating them under a mercury lamp at 30 kV for 15 min. Each sample was thoroughly checked and documented. This device was fitted with an energy-dispersive spectrum (EDS) to verify the existence of nanoparticles.

#### 4.4.3. X-ray Diffraction Analysis (XRD)

Freeze-dried powder samples of the produced ZnONPs from the leaf and fruit extracts of Citrullus colocynthis (n-n-hexane, methanol, aqueous, and ethyl acetate) were utilized for X-ray diffraction. X-ray diffraction analysis was used to investigate crystalline zinc nanoparticles (Bruker D8 advance, Bruker AXS, Billerica, MA, USA).

### 4.5. Phytochemical Analysis

#### 4.5.1. Total Flavonoid Content Determination

The total bioflavonoid concentrations were examined using the method of [60]. The extracts/samples (20 µL) were combined with 160 µL of the aqueous extract, 10 µL of the potassium acetate extract, and 10 µL of the aluminum trichloride extract in 96-well plates. The mixture was incubated at room temperature for 30 min. The optical density on a microplate reader was measured at the 405 nm wavelength. Quercetin solutions at levels of 2.5–40 µg/mL were used to construct the standard curve for the determination of complete flavonoid concentrations in equivalency to quercetin. The corresponding solvents were used as a negative control at a volume of 20 µL.

#### 4.5.2. Total Phenolic Content Determination

Using the Folin–Ciocalteu assay, the total phenol content was determined as described by [60]. The sample solutions and extracts were produced at a final concentration of 1 mg/µL. A 200 µL sample was added to a 96-well plate, followed by the addition of 90 µL of a Folin–Ciocalteu reagent with continuous stirring. Following incubation, the solution was set at 27 °C for 5 min before adding 90 µL of sodium carbonate. The resulting mix was put in an incubator for 60 min at 27 °C before measuring values at a wavelength of 630 nm. Gallic acid (3.125–25 µg/µL) was utilized to plot the standard calibration curve. The total phenol contents were reported as percent weight-to-weight gallic acid equivalents. Then, 20 µL of each of the corresponding solvents was used as a negative control.

#### 4.5.3. Total Alkaloids Determination

With a few modifications, the methodology of [61] was used to calculate the number of alkaloids. The samples/extracts were mixed in 2N HCL and then left for 30 min at 27 °C before being washed thrice with 10 mL chloroform. After bringing the pH level to neutral, a 1:1 combination of a phosphate buffer and bromocresol green (BCG) was added to this solution. Chloroform was used to extract the mixture, and the extracts were collected. The alkaloids were measured with a UV spectrometer (SHIMADZU UV-1800) at a 470 nm wavelength.

### 4.6. Biological Activities

The plant extracts of *Citrullus colocynthis* and their ZnONPs were subjected to the following bioactivity tests.

#### 4.6.1. Antibacterial Assay

Five bacterial strains—two Gram-positive strains (*Bacillus subtilis* (ATCC 6633) and *Staphylococcus aureus* (ATCC 6538)) and three Gram-negative strains (*Klebsiella pneumonia* (ATCC-1705), *Pseudomonas aeruginosa* (ATCC-15442), and *Escherichia coli* (ATCC 15224))—were used to assess the anti-bacterial potency for every sample. The well diffusion method was adopted to analyze the anti-bacterial effects of every extracted sample in vitro following the previously reported methods [62,63,64]. The bacterial strains were cultured overnight in nutrient broth. Using a spectrometer, the bacteria inoculum was adjusted to 0.5 MF (McFarland) with absorbance at 580 µm to achieve a viable count of 106 CFU per ml. Each test extract (5 µL from 20 mg/mL) was used at various concentrations preceded by a one-day incubation period at 37 °C. DMSO (5 µL) was used as the negative control, while cefixime and roxithromycin (5 µL from 4 mg/mL DMSO) were used as positive controls. Post-incubation, the zone of inhibition was measured with a Vernier caliper. All experiments were replicated thrice, and the average value was calculated with standard deviation.

The minimum amount of an antimicrobial drug needed to suppress the growth of microorganisms after a one day of incubation is known as the minimum inhibitory concentration (MIC). The samples that exhibited the strongest anti-bacterial effect with considerable inhibition zones, i.e., 12 mm, were altered to evaluate their MIC through the broth microdilution technique. Every active sample’s stock solution (40 mg/mL) was utilized to make the master plate, which included 6 mg/mL of the concentration in sterile Mueller Hinton broth (MHB) (to maintain the overall concentration of DMSO at 1%). A 96-well microtiter plate with sterile MHB was used to serially dilute samples from the master plate to achieve a resultant concentration ranging between 3.70 and 100 μg per ml. Each well was then filled with a standardized inoculum (5 × 106 CFU per ml) for every species of bacteria. The microtiter plates were incubated at 35 °C one day after being maintained at 4 °C in the refrigerator for 2 h. The MIC was defined as the smallest concentration at which the extract showed apparent inhibitory effects on growth. The assay was carried out three times.

#### 4.6.2. Antifungal Assay

The antifungal activity was analyzed using previously reported methods [62,63,64,65]. The antifungal properties of *Aspergillus fumigatus* (FFBP 66), *Mucor* species (FFBP 0300), *Fusarium solani* (FFBP 0291), and *Aspergillus flavis* (FFBP 0064) were examined. All fungi species were cultivated at 28 °C on 6.5% SDA (Sabouraud dextrose agar, pH 5.7) and refrigerated at 4 °C. Clotrimazole (4 mg/mL) was used as a positive control, and DMSO was used as a negative control. SDA plates with 25 mL of medium were inoculated with 100 L of fresh fungus. Sterilized filter paper discs (6 mm in diameter) containing test extracts (5 μL from 20 mg/mL DMSO), DMSO (5 μL), and clotrimazole (5 μL from 4 mg/mL DMSO) were placed on seeded SDA plates. The inhibition zones encircling the discs were measured in millimeters after the inoculation plates had incubated at 30 °C for 24 h.

#### 4.6.3. Brine Shrimp Cytotoxicity Assay

Brine shrimp (*Artemia salina*) eggs (Sera, Heidelberg, Germany) were produced in a small, rectangle-shaped pan (22 × 32 cm) filled with seawater. A 2 mm plastic divider with numerous perforations was placed within a big jar to divide the mixture into two unequal sections. The smaller portion was illuminated, and the eggs (approximately 25 mg) within the larger portion (which had been covered with aluminum foil) were evenly distributed. After emerging for one day, *Phototropic nauplii* (brine shrimp larvae) were separated from their shells with a separator and pipetted out from the illuminated side. The cytotoxic activity experiment was conducted on a 96-well plate containing various alphabets (A–H). In wells A and E of the microwell plate, 44 microliters of saltwater were added. In B, C, D, F, G, and H, 25 microliters of seawater were added, and 6 microliters seawater were added to A and E. From well A, 25 µL of the sample was put into well B, and from well B, 25 µL of the sample was transferred to well C. The same procedure was performed for D, and 25 µL from D was thrown away. The same procedure was followed for E, F, G, and H, and then 25 µL was disposed from H. Each microplate well received ten shrimp, and these wells were then filled with 300 µL of saltwater and left for one day. A microscope was used to examine the larvae’s survivability. This analysis was conducted thrice, and the dead larva percentage was calculated using Abbott’s methodology.

#### 4.6.4. Free Radical Scavenging Activity

The ability to scavenge free radicals was determined using the 2,2,diphenyl-1-picrylhydrazyl (DPPH) test. The 2,2,diphenyl-1-picrylhydrazyl free radical test was carried out following the methodology of [61]. About 9.6 mg of 2,2,diphenyl-1-picrylhydrazyl was dissolved in 100 mL of methanol to make a solution of 2,2,diphenyl-1-picrylhydrazyl. The tested samples were prepared at a 4 mg/mL concentration in dimethyl sulfoxide (DMSO). In DMSO, 1 mg/mL of standard ascorbate was formed. In each well of a 96-well plate, 10 µL of the test sample was added, followed by 190 µL of a 2,2,diphenyl-1-picrylhydrazyl solution. Mixtures were shaken and incubated for 60 min in the dark at 37 °C. A microplate reader was used to calculate the optical density at 515 nm. DMSO and ascorbate were used as the negative and positive controls, respectively. Each test was repeated three times, with IC50 values calculated using table curve software and the percentage inhibition calculated as follows.

% DPPH = (1 − Abs/Abc) × 100

Ac = absorbance of negative control; As = absorbance of the test sample.

### 4.7. Enzyme Inhibition Assays

#### 4.7.1. Protein Kinase Assay

According to the methodology of [65], hyphae formation takes place in the pure strain of *Streptomyces* 85E in the protein kinase assay. A bacteria lawn was cultured by spreading spores (mycelia fragments) from a fresh culture of *Streptomyces* on sterilized plates with limited ISP4 media. Sterilized 6 mm filter paper discs were dipped into 5 μL of each extract (20 mg/mL DMSO) and placed on the top of *Streptomyces* 85E-inoculated plates at a ratio of 100 g/disc. Negative and positive controls were injected in discs with DMSO and surfactin, respectively. The plates were incubated for three days at 30 °C, and the bald inhibition zone was calculated around tested samples and control discs.

#### 4.7.2. α-Amylase Inhibition Assay

The antidiabetic potential of sample extracts was evaluated using a modified version of the standard α-amylase inhibition assay [66]. A 96-well plate reaction mix consisting of 25 μL of an amylase enzyme (0.14 U/mL), 150 μL of a phosphate buffer (pH 6.8), 40 μL of a starch solution (2 milligram per liter in a potassium phosphate buffer), and 10 μL of a sample (4 milligrams per milliliter DMSO) was mixed for incubation at 323.15 K for 30 min before adding 20 μL of 1 M HCl. Following this, 90 μL of an iodine solution (5 mM iodine and 5 mM potassium iodide) was added to all individual wells. The negative control was used with no extracts of plants, blanks with no amylase, and plant extracts, and acarbose (250 μM) was used as a positive control. After incubation, absorbance was recorded at 540 nm, and the activity was measured by using the following formula:

%α-amylase inhibition = (*Os − On)*/(*Ob − On)* × 100%

*Ob* = blank well absorbance; *Os* = sample absorbance; *On =* negative control absorbance.

### 4.8. Statistical Analysis

All data are expressed as the mean ± standard deviation. A one-way ANOVA test was used for each comparison and the calculation of *p*-values, while Dunnett’s test was used for multiple comparisons. The significance level was evaluated as *p* < 0.05, *p* < 0.01, or *p* < 0.001.

## 5. Conclusions

The present study established an easy, quick, and environmentally friendly procedure to synthesize zinc oxide nanoparticles using fruit and leaf extracts of *C. colocynthis*. The physicochemical characterization of the nanoparticles revealed a particle size between ~64 nm and ~82 nm. The phytochemical assays confirmed that the complete flavonoid, phenolic, and alkaloid concentrations were higher in crude solvent extracts than in nanoparticles. All n-hexane, ethyl acetate, methanol, and aqueous plant extracts of *Citrullus colocynthis* containing ZnONPs showed significant increases in antioxidant and cytotoxic activities compared with crude extracts. However, the crude extracts showed greater maximum antimicrobial activities compared with the ZnONPs. The ZnONPs also showed increased amounts of total phenolic and flavonoid contents in comparison with the crude extracts of the plant. The crude extracts and nanoparticles of leaves showed the highest cytotoxic activity in the n-hexane solvent, with LC_50_ values 42.08 and 46.35, respectively. Potential antidiabetic activity was shown by the n-hexane (93.42%) and aqueous (82.54%) nanoparticles of the fruit.

Furthermore, the inhibition of protein kinase assay and the alpha-amylase assays revealed strong bactericidal effects and greater alpha-amylase inhibition capability, respectively, in all ZnONPs compared with crude extracts. This study indicated that the zinc oxide nanoparticles and crude extracts of *C. colocynthis* can be used as a source of pharmaceutical products and should be further investigated for biomedical studies. Most importantly, green synthesized zinc oxide nanoparticles are biologically compatible, so they cause less harm to the environment and are less toxic than nanoparticles synthesized with alternative methods. New drugs can be discovered by thoroughly investigating the separation of plant-active compounds.

## Figures and Tables

**Table 1:** Extract recovery of different parts of *C. colocynthis*.

Extract Codes	Extract Recovery			
	Leaves		Fruit	
	Total Quantity	Divisions	Total Quantity	Divisions
nH	400 mL	200/200 mL	400 ml	200/200 mL
EA	400 mL	200/200 mL	400 ml	200/200 mL
M	450 mL	225/225 mL	450 ml	225/225 mL
Aq	450 mL	225/225 mL	450 ml	225/225 mL

nH = n-hexane; EA = ethyl acetate; M = methanol; Aq = aqueous.

**Table 2:** Antibacterial activity of crude extracts and nanoparticles of leaves and fruits of *C. colocynthis*.

Extract Name	Sample	Diameter of Zone of Inhibition in mm (Mean SD) (MIC:µg/mL)									
		P.A	MIC	K.P	MIC	S.A	MIC	E.C	MIC	B.S	MIC
Leaves											
nH	C	8 ± 0.40	---	12 ± 0.36	100	6 ± 0.18	---	12 ± 0.18	100	15 ± 0.27	100
	NP	10 ± 0.25	---	39 ± 0.43 *	3.7	8 ± 0.25	---	18 ± 0.25 *	100	28 ± 0.41 *	33.3
EA	C	6 ± 0.28	---	12 ± 0.25	100	10 ± 0.3	---	13 ± 0.27	100	12 ± 0.19	100
	NP	7 ± 0.28	---	28 ± 0.19 *	3.7	7 ± 0.19	---	24 ± 0.19 *	33.3	18 ± 0.28 *	100
M	C	11 ± 0.25	---	15 ± 0.41	100	11 ± 0.6	---	13 ± 0.27	100	12 ± 0.17	100
	NP	13 ± 0.27	100	21 ± 0.48 *	100	12 ± 0.17	100	17 ± 0.65 *	100	14 ± 0.23	100
Aq	C	8 ± 0.12	---	13 ± 0.40	100	6 ± 0.45	---	28 ± 0.27	11.1	21 ± 0.45	33.3
	NP	7 ± 0.21	---	24 ± 0.32 *	33.3	12 ± 0.2 *	---	39 ± 0.43 *	3.7	34 ± 0.19 *	3.7
Fruits											
nH	C	6 ± 0.12	---	15 ± 0.56	100	4 ± 0.2	---	12 ± 0.12	100	8 ± 0.12	---
	NP	7 ± 0.25	---	21 ± 0.19 *	33.3	6 ± 0.10	---	21 ± 0.17 *	33.3	11 ± 0.12 *	---
EA	C	5 ± 0.18	---	12 ± 0.32	100	8 ± 0.25	---	13 ± 0.27	100	6 ± 0.7	---
	NP	5 ± 0.18	---	18 ± 0.25 *	100	8 ± 0.25	---	17 ± 0.65^*^	100	8 ± 0.6	---
M	C	8 ± 0.35	---	12 ± 0.30	100	6 ± 0.32	---	12 ± 0.21	100	12 ± 0.51	100
	NP	12 ± 0.2 *	100	13 ± 0.18	100	8 ± 0.35	---	25 ± 0.6 *	33.3	21 ± 0.32 *	33.3
Aq	C	9 ± 0.41	---	16 ± 0.21	100	5 ± 0.62	---	12 ± 0.12	100	15 ± 0.25	100
	NP	13 ± 0.2 *	100	17 ± 0.21	100	5 ± 0.62	---	14 ± 0.8	100	25 ± 0.21 *	33.3
Controls											
Cef.		22 ± 0.88	1.11	20 ± 0.12	1.11	***	***	20 ± 1.5	3.33	***	***
Rox.		***	***	***	***	23 ± 0.54	1.11	***	***	23 ± 0.54	1.11
DMSO		----	----	---	---	---	---	---	---	---	---

Sample concentration = 100 µg per disc. Values (mean ± SD) = average of triplicate analysis of each plant extract (n value of 1 × 3). --- = no activity. Samples showing zone of inhibition ≥12 mm were not applicable for MIC determination. S.A = *Staphylococcus aureus*; B.S = *Bacillus subtilis*; P.A = *Pseudomonas aeruginosa*; K.P = *Klebsiella pneumoniae*; E.C = *Escherichia coli.* Values with superscript (*) letters show significantly (*p* < 0.05) different means. Values with superscript (***) letters show significantly (*p* < 0.001) different means. C = negative control, which is the crude plant extract in a respective solvent; NP = biogenic nanoparticles.

**Table 3:** Antifungal activity of crude extracts and nanoparticles of leaves and fruits of *C. colocynthis*.

Plant Part	Extracts	Samples	Antifungal Activity			
			Zone of Inhibition (mm)			
			*A. flavus*	*A. fumigatus*	*Mucor* sp.	*F. solani*
Leaves	n-Hexane	NP	---	---	---	---
		C	---	---	---	7 ± 0.02
	Ethyl acetate	NP	---	---	---	7 ± 0.02 *
		C	---	---	---	---
	Methanol	NP	---	7 ± 0.03 *	---	12 ± 0.01
		C	---	---	---	9 ± 0.04
	Aqueous	NP	7 ± 0.01 *	---	---	11 ± 0.02
		C	---	---	---	10 ± 0.01
Fruits	n-Hexane	NP	---	11 ± 0.01	---	11 ± 0.03
		C	---	10 ± 0.02	---	13 ± 0.02
	Ethyl acetate	NP	---	11 ± 0.06	---	12 ± 0.01
		C	---	10 ± 0.02	---	12 ± 0.01
	Methanol	NP	7 ± 0.02 *	13 ± 0.01	---	11 ± 0.02
		C	---	12 ± 0.05	---	13 ± 0.01
	Aqueous	NP	---	12 ± 0.12	---	13 ± 0.08
		C	---	11 ± 0.09	---	11 ± 0.09
Positive Controls	Clotrim.		20 ± 0.01	24 ± 0.03	27 ± 0.02	28 ± 0.01
	DMSO		---	---	---	---

Values (mean ± SD) = average of triplicate of each test sample (n value of 1 × 3). --- Value of No activity in the disc diffusion assay; Clotrim = clotrimazole. Values with superscript (*) letters show significantly (*p* < 0.05) different means. C = negative control, the crude plant extract in a respective solvent; NP = biogenic nanoparticles.

**Table 4:** Cytotoxicity potential of crude extracts and nanoparticles of leaves and fruits of *C. colocynthis*.

Plant Parts	Extracts	Samples	Percent Mortality ± STD (Conc. in µg/mL)			
			200	100	50	LC_50_
CC leaves	n-Hexane	NP	50 ± 1.50 *	60 ± 1.00 *	60 ± 1.00	46.84
		C	100 ± 1.50	100 ± 1.50	60 ± 1.40	42.41
	Ethyl acetate	NP	60 ± 1.50	40 ± 1.00 *	10 ± 1.50 *	110.45
		C	80 ± 1.50	80 ± 1.50	60 ± 1.70	72.92
	Methanol	NP	60 ±1.25	10 ± 1.60	10 ± 1.40	150.6
		C	40 ± 0.85	20 ± 0.25	---	>200
	Aqueous	NP	20 ± 1.5	20 ± 1.50	---	>200
		C	20 ± 1.75	10 ± 1.50	---	>200
CC fruit	n-Hexane	NP	40 ± 1.50 *	40 ± 1.50	10 ± 1.40	130.5
		C	70 ± 1.5	30 ± 1.0	10 ± 1.30	140.6
	Ethyl acetate	NP	10 ± 1.50 *	10 ± 1.00	60 ± 1.00	146
		C	60 ± 1.2	20 ± 1.5	10 ± 1.60	170.6
	Methanol	NP	70 ± 1.00	30 ± 1.50 *	10 ± 1.70 *	185.8
		C	70 ± 1.15	60 ± 1.25	40 ± 1.00	>200
	Aqueous	NP	---	---	---	---
		C	30 ± 1.50	10 ± 1.00	---	>200

* LC50 of doxorubicin (positive control employed in the brine shrimp lethality assay) was 5.93 µg/mL. DMSO was applied as a negative control, and the values are expressed as the mean of triplicate ± SD. Values with superscript (*) letters show significantly (*p* < 0.05) different means. C = negative control, the crude plant extract in a respective solvent; NP = biogenic nanoparticles.

**Table 5:** Protein kinase inhibition of crude extracts and nanoparticles of leaves and fruits of *C. colocynthis*.

Plant Parts	Extracts	Samples	Numerical Value	Activity
Leaves	n-Hexane	NP	11	Clear
		C	5	NA
	Ethyl acetate	NP	78	Clear
		C	12	Clear
	Methanol	NP	28	Clear\Bald
		C	17	Bald
	Aqueous	NP	10	Clear
		C	35	Clear
Fruits	n-Hexane	NP	49	Bald
		C	5	NA
	Ethyl acetate	NP	28	Clear
		C	5	NA
	Methanol	NP	48	Clear
		C	64	Bald
	Aqueous	NP	19	Bald
		C	15	Clear

DMSO: negative control; surfactin: positive control (20 µg/disc; 16 mm zone). C = negative control, the crude plant extract in a respective solvent; NP = biogenic nanoparticles.

## Data Availability

Not applicable.

## References

[1] Selim Y.A., Azb M.A., Ragab I., Abd El-Azim M.H.M. (2020). Green Synthesis of Zinc Oxide Nanoparticles Using Aqueous Extract of Deverra tortuosa and their Cytotoxic Activities. Sci. Rep..

[2] Li Y., Chen S.M., Ali M.A., Al-Hemaid B. (2013). Biosynthesis and electrochemical characterization of silver nanoparticles from leaf extract of *(Adenium obesum)* and its application to antibacterial effect. Int. J. Electrochem. Sci..

[3] Geonmonond R., da Silva A., Camargo P.H.C. (2018). Controlled synthesis of noble metal nanomaterials: Motivation, principles, and opportunities in nanocatalysis. An. Acad. Bras. Ciênc..

[4] Hemanth K.N., Andia J.D., Manjunatha S., Murali M., Amruthesh K.N., Jagannath S. (2019). Antimitotic and DNA-binding potential of biosynthesized ZnO-NPs from leaf extract of *Justicia wynaadensis* (Nees) Heyne—A medicinal herb. Biocatal. Agric. Biotechnol..

[5] Murali M., Mahendra C., Nagabhushan, Rajashekar N., Sudarshana M., Raveesha K., Amruthesh K. (2017). Antibacterial and antioxidant properties of biosynthesized zinc oxide nanoparticles from *Ceropegia candelabrum* L.—An endemic species. Spectrochim. Acta A Mol. Biomol. Spectrosc..

[6] Somu P., Paul S. (2019). Protein assisted one pot controlled synthesis of monodispersed and multifunctional colloidal silver-gold alloy nanoparticles. J. Mol. Liq..

[7] Simon S., Sibuyi N.R.S., Fadaka A.O., Meyer S., Josephs J., Onani M.O., Meyer M., Madiehe A.M. (2022). Biomedical Applications of Plant Extract-Synthesized Silver Nanoparticles. Biomedicines.

[8] Rahuman H.B.H., Dhandapani R., Narayanan S., Palanivel V., Paramasivam R., Subbarayalu R., Thangavelu S., Muthupandian S. (2022). Medicinal plants mediated the green synthesis of silver nanoparticles and their biomedical applications. IET Nanobiotechnol..

[9] Anandan S., Mahadevamurthy M., Ansari M.A., Alzohairy M.A., Alomary M.N., Farha S.S., Sarjan H.N., Mahendra C., Lakshmeesha T.R., Hemanth K.N.K. (2019). Biosynthesized ZnO-NPs from *Morus indica* attenuates methylglyoxal-induced protein glycation and RBC damage: In-vitro, in-vivo, and molecular docking study. Biomolecules.

[10] Kumar N.K.H., Murali M., Satish A., Singh S.B., Gowtham H.G., Mahesh H.M., Lakshmeesha T.R., Amruthesh K.N., Jagannath S. (2020). Bioactive and Biocompatible Nature of Green Synthesized Zinc Oxide Nanoparticles from Simarouba glauca DC.: An Endemic Plant to Western Ghats, India. J. Clust. Sci..

[11] Thatoi P., Kerry R.G., Gouda S., Das G., Pramanik K., Thatoi H., Patra J.K. (2016). Photo-mediated green synthesis of silver and zinc oxide nanoparticles using aqueous extracts of two mangrove plant species, *Heritiera fomes* and *Sonneratia apetala* and investigation of their biomedical applications. J. Photochem. Photobiol. B Biol..

[12] Ansari M.A., Murali M., Prasad D., Alzohairy M.A., Almatroudi A., Alomary M.N., Udayashankar A.C., Singh S.B., Asiri S.M.M., Ashwini B.S. (2020). Cinnamomum verum Bark Extract Mediated Green Synthesis of ZnO Nanoparticles and Their Antibacterial Potentiality. Biomolecules.

[13] Prasad K.S., Prasad S.K., Ansari M.A., Alzohairy M.A., Alomary M.N., Alyahya S., Srinivasa C., Murali M., Ankegowda V.M., Shivamallu C. (2020). Tumoricidal and Bactericidal Properties of ZnONPs Synthesized Using *Cassia auriculata* Leaf Extract. Biomolecules.

[14] Chunchegowda U.A., Shivaram A.B., Mahadevamurthy M., Ramachndrappa L.T., Lalitha S.G., Krishnappa H.K.N., Anandan S., Sudarshana B.S., Chanappa E.G., Ramachandrappa N.S. (2020). Biosynthesis of Zinc Oxide Nanoparticles Using Leaf Extract of Passiflora subpeltata: Characterization and Antibacterial Activity Against Escherichia coli Isolated from Poultry Faeces. J. Clust. Sci..

[15] Faisal S., Jan H., Shah S.A., Shah S., Khan A., Akbar M.T., Rizwan M., Jan F., Wajidullah, Akhtar N. (2021). Green Synthesis of Zinc Oxide (ZnO) Nanoparticles Using Aqueous Fruit Extracts of *Myristica fragrans*: Their Characterizations and Biological and Environmental Applications. ACS Omega.

[16] Agarwal H., Shanmugam V.K. (2019). Synthesis and optimization of zinc oxide nanoparticles using Kalanchoe pinnata towards the evaluation of its anti-inflammatory activity. J. Drug Deliv. Sci. Technol..

[17] Ali S.G., Ansari M.A., Jamal Q.M.S., Almatroudi A., Alzohairy M.A., Alomary M.N., Rehman S., Mahadevamurthy M., Jalal M., Khan H.M. (2021). *Butea monosperma* seed extract mediated biosynthesis of ZnO NPs and their antibacterial, antibiofilm and anti-quorum sensing potentialities. Arab. J. Chem..

[18] Matinise N., Fuku X.G., Kaviyarasu K., Mayedwa N., Maaza M. (2017). Applied Surface Science ZnO nanoparticles via Moringa oleifera green synthesis: Physical properties & mechanism of formation. Appl. Surf. Sci..

[19] Ahmed S., Ahmad M., Swami B.L., Ikram S. (2015). A review on plants extract mediated synthesis of silver nanoparticles for antimicrobial applications: A green expertise. J. Adv. Res..

[20] Ghorbanpour M.M., Mazloumi M., Nouri A., Lotfiman A. (2017). Silver-doped Nano clay with antibacterial activity. J. Ultrafine Grained Nanostruct. Mater..

[21] Somu P., Paul S. (2018). Casein based biogenic-synthesized zinc oxide nanoparticles simultaneously decontaminate heavy metals, dyes, and pathogenic microbes: A rational strategy for wastewater treatment. J. Chem. Technol. Biotechnol..

[22] Albayaty N. (2011). The most medicinal plants used in Iraq. Traditional knowledge. Advances in Environmental Biology.

[23] Bourhia M., Bouothmany K., Bakrim H., Hadrach S., Salamatullah A.M., Alzahrani A., Khalil A.H., Albadr N.A., Gmouh S., Laglaoui A. (2021). Chemical Profiling, Antioxidant, Antiproliferative, and Antibacterial Potentials of Chemically Characterized Extract of *Citrullus colocynthis* L. Seeds. Separations.

[24] Khare C.P. (2007). Indian Medicinal Planta—An Illustrated Dictionary.

[25] Marzouk B., Marzouk Z., Fenina N., Bouraoui A., Aouni M. (2011). Anti-inflammatory and analgesic activities of Tunisian *Citrullus colocynthis* Schrad. immature fruit and seed organic extracts. Eur. Rev. Med. Pharmacol. Sci..

[26] Murali M., Anandan S., Ansari M., Alzohairy M., Alomary M., Asiri S., Almatroudi A., Thriveni M., Singh S., Gowtham H. (2021). Genotoxic and Cytotoxic Properties of Zinc Oxide Nanoparticles Phyto-Fabricated from the Obscure Morning Glory Plant *Ipomoea obscura* (L.) Ker Gawl. Molecules.

[27] Ahmed S., Saifullah, Ahmad M., Swami B.L., Ikram S. (2016). Green synthesis of silver nanoparticles using *Azadirachta indica* aqueous leaf extract. J. Radiat. Res. Appl. Sci..

[28] Meybodi M.S. (2020). A review on pharmacological activities of *Citrullus colocynthis* (L.) Schrad. Asian J. Res. Rep. Endocrinol..

[29] Tannin-Spitz T., Bergman M., Grossman S. (2007). Cucurbitacin glucosides: Antioxidant and free-radical scavenging activities. Biochem. Biophys. Res. Commun..

[30] Duangmano S., Dakeng S., Jiratchariyakul W., Suksamrarn A., Smith D.R., Patmasiriwat P. (2010). Antiproliferative effects of cucurbitacin B in breast cancer cells down-regulation of the c-Myc/hTERT/telomerase pathway and obstruction of the cell cycle. Int. J. Mol. Sci..

[31] Christensen L., Vivekanandhan S., Misra M., Mohanty A.K. (2011). Biosynthesis of silver nanoparticles using murraya koenigii (curry leaf): An investigation on the effect of broth concentration in reduction mechanism and particle size. Adv. Mater. Lett..

[32] Zak A.K., Majid W.A., Mahmoudian M., Darroudi M., Yousefi R. (2013). Starch-stabilized synthesis of ZnO nanopowders at low temperature and optical properties study. Adv. Powder Technol..

[33] Jiang J., Pi J., Cai J. (2018). The Advancing of Zinc Oxide Nanoparticles for Biomedical Applications. Bioinorg. Chem. Appl..

[34] Sirelkhatim A., Mahmud S., Seeni A., Kaus N.H.M., Ann L.C., Bakhori S.K.M., Hasan H., Mohamad D. (2015). Review on Zinc Oxide Nanoparticles: Antibacterial Activity and Toxicity Mechanism. Nano-Micro Lett..

[35] Saraniyadevi J., Bhimba V. (2012). In-vitro anticancer activity of silver nanoparticles synthesized using the extract of (*Gelidiella*) species. Int. J. Pharm..

[36] Mahendra C., Chandra M.N., Murali M., Abhilash M., Singh S.B., Satish S., Sudarshana M. (2020). Phyto-fabricated ZnO nanoparticles from *Canthium dicoccum* (L.) for antimicrobial, anti-tuberculosis and antioxidant activity. Process Biochem..

[37] Siddhuraju P., Becker K. (2003). Antioxidant properties of various solvent extracts of total phenolic constituents from three die rent agro climatic origins of drumstick tree (*Moringa oleifera* Lam.) leaves. J. Agric. Food Chem..

[38] Soobrattee M.A., Neergheen V.S., Luximon-Ramma A., Aruoma O.I., Bahorun T. (2005). Phenolics as potential antioxidant therapeutic agents: Mechanism and actions. Mutat. Res./Fund. Mol. Mech. Mutagen..

[39] Adebooye O.C., Vijayalakshmi R., Singh V. (2008). Peroxidase activity, chlorophylls, and antioxidant profile of two leaf vegetables (*Solanum nigrum* L. and *Amaranthus cruentus* L.) under six pretreatment methods before cooking. Int. J. Food Sci. Technol..

[40] Yen G.C., Chuang D.Y. (2000). Antioxidant properties of water extracts from (*Cassia tora* L.) in relation to the degree of roasting. J. Agric. Food Chem..

[41] Uddin M.K., Juraimi A.S., Ali M.E., Ismail M.R. (2012). Evaluation of antioxidant properties and mineral composition of purslane (*Portulaca oleracea* L.) at die rent growth stages. Int. J. Mol. Sci..

[42] Burri S.C.M., Ekholm A., Håkansson Å., Tornberg E., Rumpunen K. (2017). Antioxidant capacity and major phenol compounds of horticultural plant materials not usually used. J. Funct. Foods.

[43] Panche A.N., Diwan A.D., Chandra S.R. (2020). Flavonoids: An overview. J. Nutr. Sci..

[44] Wang L., Weller C.L. (2006). Recent advances in extraction of nutraceuticals from plants. Trends Food Sci. Technol..

[45] Shrestha P.M., Dhillion S.S. (2006). Diversity and Traditional Knowledge Concerning Wild Food Species in a Locally Managed Forest in Nepal. Agrofor. Syst..

[46] Sharmila G., Muthukumaran C., Sandiya K., Santhiya S., Pradeep R.S., Kumar N.M., Suriyanarayanan N., Thirumarimurugan M. (2018). Biosynthesis, characterization, and antibacterial activity of zinc oxide nanoparticles derived from *Bauhinia tomentose* leaf extract. J. Nanostruct. Chem..

[47] Azizi S., Mohamad R., Mahdavi Shahri M. (2017). Green Microwave-Assisted Combustion Synthesis of Zinc Oxide Nanoparticles with *Citrullus colocynthis* (L.) Schrad: Characterization and Biomedical Applications. Molecules.

[48] Siripireddy B., Mandal B.K. (2017). Facile green synthesis of zinc oxide nanoparticles by Eucalyptus globulus and their photocatalytic and antioxidant activity. Adv. Powder Technol..

[49] Alamdari S., Ghamsari M.S., Lee C., Han W., Park H.-H., Tafreshi M.J., Afarideh H., Ara M.H.M. (2020). Preparation and Characterization of Zinc Oxide Nanoparticles Using Leaf Extract of *Sambucus ebulus*. Appl. Sci..

[50] Sadighara A., Parisa S., Moghadam A.J., Golamreza J., Nabi S., Akbar A.L. (2013). Potential therapeutic effects of (*Morus alba*) leaf extract on modulation oxidative damages induced by hyperglycemia in cultured fetus fibroblast cells. Glob. Vet..

[51] Ghagane S.C., Puranik S.I., Kumbar V.M., Nerli R.B., Jalalpure S.S., Hiremath M.B. (2017). In-vitro antioxidant and anticancer activity of (*Lee indica*) leaf extracts on human prostate cancer cell lines. Integr. Med. Res..

[52] Fukumoto L.R., Mazza G. (2000). Assessing Antioxidant and Prooxidant Activities of Phenolic Compounds. J. Agric. Food Chem..

[53] Khan Z.U.H., Sadiq H.M., Shah N.S., Khan A.U., Muhammad N., Hassan S.U., Tahir K., Safi S.Z., Khan F.U., Imran M. (2019). Greener synthesis of zinc oxide nanoparticles using Trianthema portulacastrum extract and evaluation of its photocatalytic and biological applications. J. Photochem. Photobiol. B Biol..

[54] Mayerhof E.R., Koncz-Kalman R.Z., Nawrath C., Bakkeren G., Crameri A., Angelis K., Redei G.P., Schell J.B., Hohn K.J. (2009). T-DNA integration a mode of illegitimate recombination inplants. EMBO J..

[55] Bisht G., Rayamajhi S. (2016). ZnO Nanoparticles: A Promising Anticancer Agent. Nanobiomedicine.

[56] Gunalana S., Sivaraja R., Rajendran V. (2012). Green synthesized ZnO nanoparticles against bacterial and fungal pathogens. Prog. Nat. Sci..

[57] Pal S., Tak Y.K., Song J.M. (2007). Does the Antibacterial Activity of Silver Nanoparticles Depend on the Shape of the Nanoparticle? A Study of the Gram-Negative Bacterium *Escherichia coli*. Appl. Environ. Microbiol..

[58] Santosh V., Thakkar S., Kevin M., Allegre S.B., Joshi D.B., Volkin C., Russell M. (2012). An application of ultraviolet spectroscopy to study interactions in proteins solutions at high concentrations. Pharm. Sci..

[59] Schaffer B., Hohenester U., Trügler A., Hofer F. (2009). High-resolution surface plasmon imaging of gold nanoparticles by energy-filtered transmission electron microscopy. Phys. Rev. B.

[60] Ul-Haq I., Ullah N., Bibi G., Kanwal S., Ahmad M.S., Mirza B. (2012). Antioxidant and Cytotoxic Activities and Phytochemical Analysis of *Euphorbia wallichii* Root Extract and its Fractions. Iran. J. Pharm. Res..

[61] Ajanal M., Gundkalle M.B., Nayak S.U. (2012). Estimation of total alkaloid in Chitrakadivati by UV-Spectrophotometer. Anc. Sci. Life.

[62] Zahra S.S., Ahmed M., Qasim M., Gul B., Zia M., Mirza B. (2017). Polarity-based characterization of biologically active extracts of *Ajuga bracteosa* Wall ex. Benth and RP-HPLC analysis. BMC Complement. Altern. Med..

[63] Kebede T., Gadisa E., Tufa A. (2021). Antimicrobial activities evaluation and phytochemical screening of some selected medic-inal plants: A possible alternative in the treatment of multidrug-resistant microbes. PLoS ONE.

[64] Hameed B., Ali Q., Hafeez M.M., Malik A. (2020). Antibacterial and antifungal activity of fruit, seed and root extracts of *Citrullus colocynthis* plant. Biol. Clin. Sci. Res. J..

[65] Fatima H., Khan K., Zia M., Ur-Rehman T., Mirza B., Haq I.-U. (2015). Extraction optimization of medicinally important metabolites from Datura innoxia Mill.: An in vitro biological and phytochemical investigation. BMC Complement. Altern. Med..

[66] Kim J.S., Kwon C.S., Sou K.H. (2000). Inhibition of alpha glycosidase and amylase luteolin a flavonoid. Biosci. Biotechnol. Biochem..

